# Innovation in Urology: Three Dimensional Printing and Its Clinical Application

**DOI:** 10.3389/fsurg.2020.00029

**Published:** 2020-06-02

**Authors:** David A. P. Mathews, Andrew Baird, Marc Lucky

**Affiliations:** ^1^University Hospital Coventry & Warwickshire, Coventry, United Kingdom; ^2^Aintree University Hospital, Liverpool, United Kingdom

**Keywords:** additive manufacturing, stereolithography, urology, surgery, three-dimensional, printing

## Abstract

Three-dimensional (3D) printing allows rapid prototyping of novel equipment as well as the translation of medical imaging into tangible replicas of patient-specific anatomy. The technology has emerged as a versatile medium for innovation in medicine but with ever-expanding potential uses, does 3D printing represent a valuable adjunct to urological practice? We present a concise systematic review of articles on 3D printing within urology, outlining proposed benefits and the limitations in evidence supporting its utility. We review publications prior to December 2019 using guidelines outlined by the Preferred Reporting Items for Systematic Reviews and Meta-Analysis (PRISMA) statement. Of 117 identified articles, 67 are included highlighting key areas of research as the use of patient-specific models for patient education, surgical planning, and surgical training. Further novel applications included printed surgical tools, patient-specific surgical guides, and bioprinting of graft tissues. We conclude to justify its adoption within standard practice, further research is required demonstrating that use of 3D printing can produce; direct and measurable improvements in patient experience, consistent evidence of superior surgical outcomes or simulation which surpasses existing means' both in fidelity and enhancement of surgical skills. Although exploration of 3D printing's urological applications remains nascent, the seemingly limitless scope for innovation and collaborative design afforded by the technology presents undeniable value as a resource and assures a place at the forefront of future advances.

## Introduction

Stereolithography or additive manufacturing, describes forming three-dimensional (3D) objects by step-wise layered addition of material. 3D printing allows successive production of structurally distinct objects instead of the mass production of identical items typically achieved by subtractive manufacturing (1).

This underpins the primary rationale for 3D printings' increasing utilization within urology: As every patient is unique, both their surgical treatment and adjuncts to it should be similarly individualized. 3D printing can utilize data from medical imaging to produce structures customized from and for a patients' individual anatomy (2). Furthermore, products can be functionally tested then quickly adapted in the next iteration.

Three-dimensional printing has the potential to enhance patient-specificity of pre-operative counseling, surgical simulation, implantable prostheses and even transplant organs (3). Customizable surgical instruments could be shared digitally, adjusted to preference and produced as required (3, 4). However, with printers used in surgical research ranging in price from $2000 to $900,000 this resource requires investment (5). Maintenance, cartridge consumables, specialist software for converting medical imaging into printable format and computer hardware further add to setup costs.

**Figure 1:**
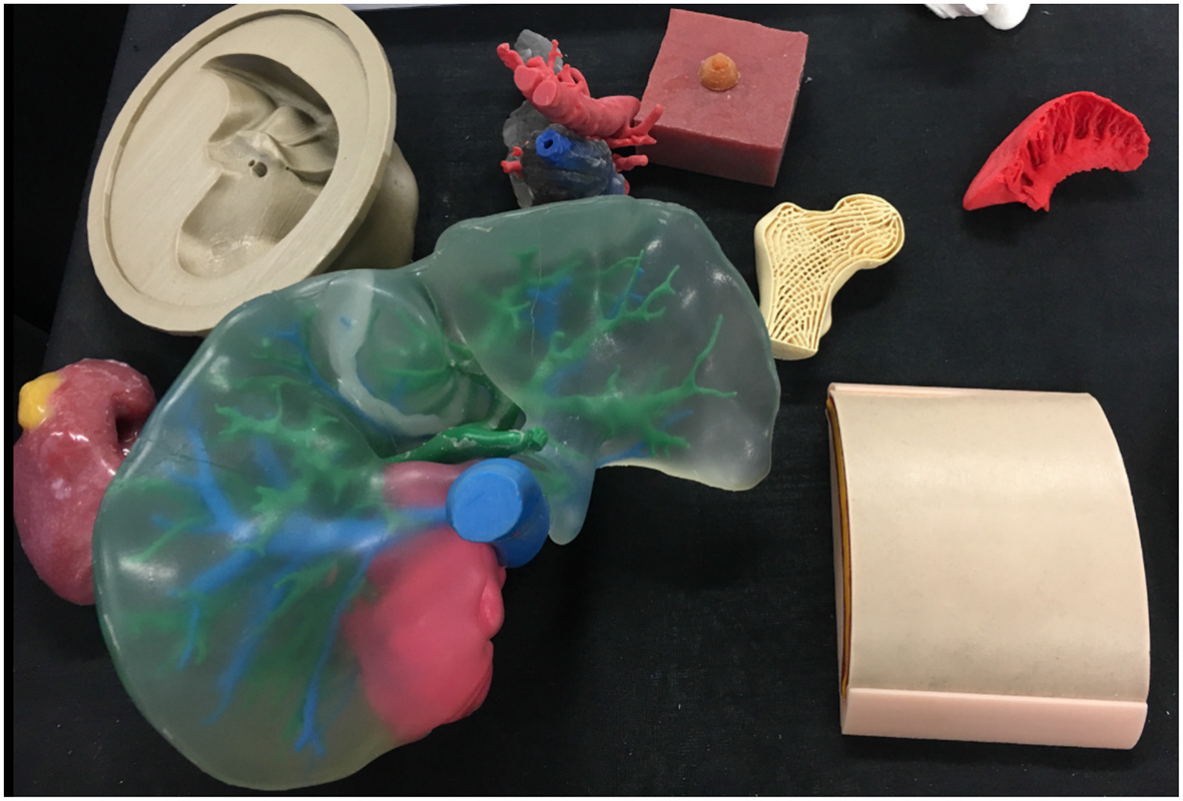
A selection of models made via 3D printing or 3D printed molds including a renal tumor model (left), liver (center), femoral head (right), and skin (right).

**Figure 2:**
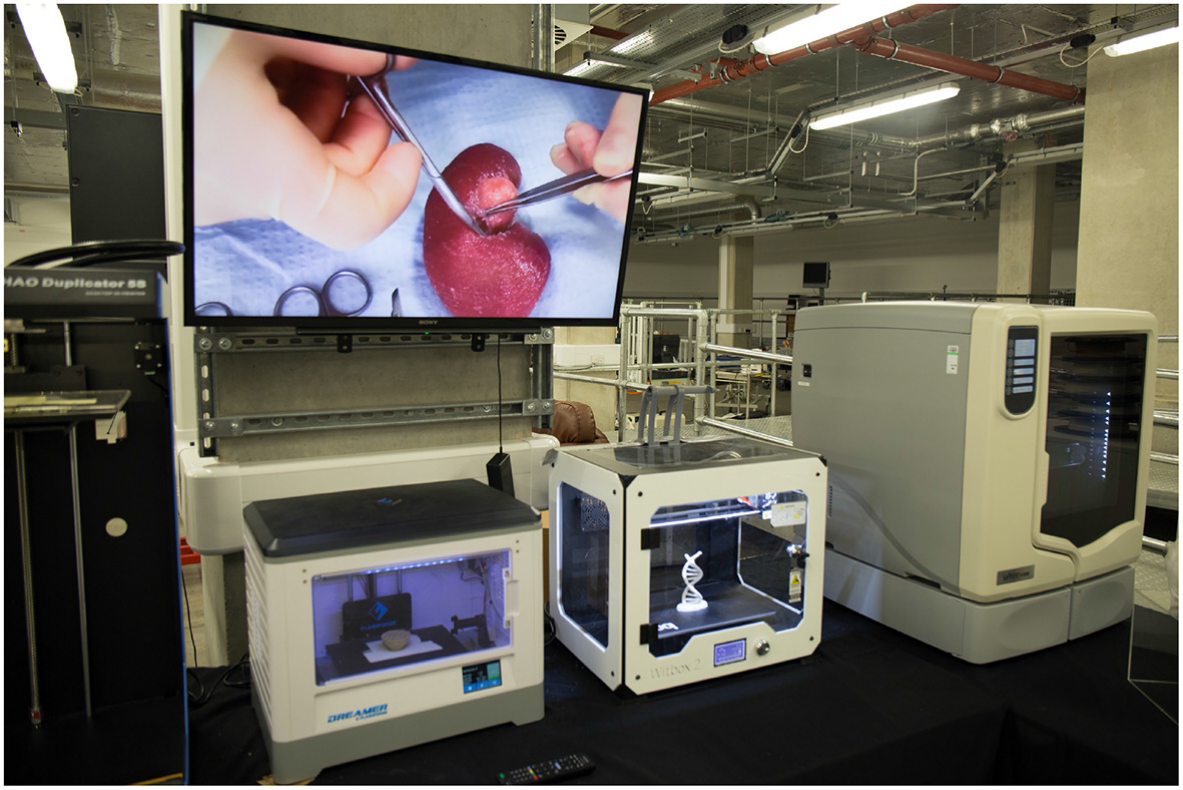
A selection of three-dimensional printers in use at 3D LifePrints hub at Alder Hey Childrens Hospital, Liverpool.

With current literature detailing diverse urological applications, distinguishing potential from proven advantage is key to guiding future practice and research alike. We therefore aim to elucidate 3D printing's confirmed benefits to patient and clinician whilst highlighting those requiring further evidence. Please refer to the Supplementary Material for details on review methodology (6). Figures 1 and 2 Illustrate products of 3D printing and a range of 3D printers.

## Patient Education

Coupling medical 3D imaging with additive manufacturing allows accurate modeling of individual patients' anatomy by converting DICOM (digital imaging and communications in medicine) data to stereolithography (STL) format (2). Bernard created kidney models with transparent resin for renal parenchyma showing tumor, vasculature, and collecting system in patients awaiting partial nephrectomy. These reportedly improved patients understanding of kidney physiology, anatomy, tumor characteristics, and the planned procedure (7). Altahay used 3D-printed models prior to percutaneous nephrolithotomy (PCNL), reporting similar improvements in patient understanding as assessed by questionnaires before and after counseling with the 3D model (8).

Teishima demonstrated superior questionnaire-assessed understanding in both patients and family members prior to partial nephrectomy when comparing explanation with 3D-printed models to using computerized tomography (CT) alone (9). Whereas Schmit reported no statistically significant improvement in patient understanding from explanation of cryoablation with 3D models compared to 2D imaging after correcting for different counseling physicians (10).

In patients undergoing partial nephrectomy or radical prostatectomy, Wake et al. compared counseling with standard imaging to imaging plus either a 3D printed model, augmented reality model or 3D computer model (11). Patient understanding was assessed via Likert scale survey with 3D-printed models and 3D computer models receiving higher scores than the control group. Only the 3D-printed model showed statistically significant improvements, including in patient-rated comfort with the surgical plan thus establishing a link between patients understanding and anxiety. A summary of reviewed articles relating to patient education is provided in Table 1.

**Table 1:** Articles relating to patient education−5 papers.

**References**	**Paper type and case number**	**Area of urology**	**Application**	**Reported outcomes and limitations**	**Cost and time to produce model**
Wake et al. (2)	Case series (*n* = 127)	Prostate Cancer and Renal Cancer	Radical prostatectomy and partial nephrectomy	Improved 5-point Likert scale survey on understanding of disease and surgical procedure Patients were counseled twice if using a 3D adjunct	Not reported
Schmit et al. (10)	Pilot study (*n* = 25)	Renal Cancer	Renal cryoablation	No significant improvement in patient anatomical or procedural knowledge compared to control High perceived value by patients	$400 40 h
Teishima et al. (9)	Case series (*n* = 29)	Renal cancer	Partial nephrectomy	Improved understanding of anatomy and tumor relationships for patient and family. Procedure understanding improved only in patient.	Not reported
Atalay et al. (8)	Pilot study (*n* = 5)	Renal stones	PCNL	Improved questionnaire scores on understanding of kidney anatomy, stone position and surgical procedure No control group. Duplicated explanation with 3D-model	$100 2 h
Bernard et al. (7)	Pilot study (*n* = 7)	Renal cancer	Partial nephrectomy	Improved questionnaire-assessed understanding of physiology, anatomy, tumor characteristics and planned surgical procedure No control group. Duplicated explanation with 3D-model	$560

## Surgical Planning

In Zhang's study, models of patients' kidneys with T1 tumors received positive face validation scores by experienced Urologists for representation of tumor size and inter-related structures but details of renal vasculature and the collecting system were scored less favorably (12). Silberstein presented cases of T1 renal tumors where 3D-printed models were used as a real-time reference for surgeons when performing reconstruction and assessing resection in relation to the hilar vessels and collecting system (13). Surgical outcomes were reported but with no control arm for comparison. Komai reported outcomes for partial nephrectomies with nephrometry scores ≥8 where a “4D” printed model was used in surgical planning (14). The proposed 4th dimension was time as the model included a removable tumor with a 2–5 mm margin to assess the defect in relation to renal structures but similarly no control arm was included to indicate superior outcomes.

Wake et al demonstrated 3D models could (a) change experienced urologist's planned surgical approach for treatment of a renal tumor and (b) the planned approach based on a 3D-printed model was more likely to be followed than one based on 2D imaging. (15).

Maddox used patient-specific models to simulate robot-assisted partial nephrectomies prior to the actual procedure and outcomes were compared to the Tulane Urology prospectively maintained database. Cases using the surgical models had larger tumors (4.3 vs. 3.4 cm, *p* = 0.4), fewer complications (0 vs. 20%), longer warm ischemic time (25 vs. 21.6 min, *p* = 0.9), fewer positive margins (0 vs. 7.4%) and shorter hospitalization time (1.86 vs. 2.4 days, *p*−0.12). The only statistically significant difference however was a lower estimated blood loss (185.7 vs. 235.6 ml, *p* = 0.01) (16). Kyung et al. compared the outcomes for partial nephrectomy aided by prior inspection of a 3D-printed model against a control cohort and similarly the only significant difference was reduced estimated blood loss (17) whereas, Fan reported a significant reduction in warm ischemic time (18).

Twelve studies described using 3D-printing in prostate cancer diagnosis and management but only 2 addressed patient-tailored pre-operative planning; Shin used pre-biopsy prostatic MRI's of patients to construct translucent models of the prostate including the biopsy-proven malignant lesion and neurovascular bundles (NVB). The relationship between prostate, lesion and NVB was assessed using these before performing nerve-sparing radical robotic prostatectomy. A wider (1 mm) area of periprostatic tissue was dissected at regions high risk for extra-capsular extension based on the models. Although high risk cases (pT2c [*n* = 1], pT3a [*n* = 2], and pT3b [*n* = 2]), histopathology confirmed all having negative surgical margins (19). Wang used translucent prostate models with visible MRI-identified lesions to aid cognitive prostate biopsy. Standard systematic biopsies were taken, followed by 2–3 targeted biopsies. A higher positive biopsy rate was reported in the targeted biopsies and higher-grade disease was identified (20).

We identified 3 studies using 3D-printing for patient-specific aids to percutaneous nephrolithotomy (PCNL). Atalay constructed anatomically accurate models of patients with complex staghorn calculi and presented these to surgical residents before PCNL. Their understanding of the collecting system anatomy, stone location and optimal entry calyx was assessed via questionnaire with reported improvement in all categories (21). Xu created copies of patients renal anatomy, containing 3D-printed facsimiles of their stones and simulated the procedure using different puncture sites to determine the optimal approach. CT-assessed stone clearance with the models correlated well with procedures using the same puncture sites (22). Golab used a virtual 3D model of a patient with a horseshoe kidney and bilateral stones to determine the optimal puncture site and angle for PCNL, then printed a sterilisable surgical guide (23). The guide was positioned using vertebral spinous processes as markers and achieving nephroscopic access in this challenging case reportedly took 3 min.

This method of surgical guide has been utilized to implant tined leads for sacral nerve modulation with Zhang et al. reporting reduced punctures, procedural time and fluoroscopy use (24). A statistically significant reduction in effective voltage after using the surgical guide also indicated a more optimally positioned lead. An expert consensus guideline by Shaito and Ye detail potential use of 3D-printed templates for brachytherapy seed implantation (25). A summary of reviewed articles relating to surgical planning is provided in Table 2.

**Table 2:** Articles relating to surgical planning−21 papers.

**References**	**Paper type and case number**	**Area of urology**	**Application**	**Reported outcomes and limitations**	**Cost and time to produce model**
**SURGICAL PLANNING: RENAL CANCER – 12 PAPERS**					
Fan et al. (20)	Retrospective series (*n* = 127)	Renal cancer	Partial nephrectomy	Statistically significant reduction in warm ischemia time and increase in surgery waiting time compared to control arm	Not reported
Kyung et al. (17)	Case series (*n* = 17)	Renal cancer	Partial nephrectomy	Statistically significant reduction in estimated blood loss compared to control arm	$600 5 days
Glybochko et al. (34)	Pilot study (*n* = 5)	Renal cancer	Partial and Radical Nephrectomy	Simulation with patient-specific model changed surgeons planned approach compared to CT imaging alone No control arm for comparison (34)	$150-450
Libby and Silberstein (35)	Case report (*n* = 1)	Renal cancer	Radical Nephrectomy	Scrutiny of model reportedly obviated need for bypass and influenced surgical plan and approach No quantifiable measures of benefit (35)	Not reported
Maddox et al. (16)	Feasibility study (*n* = 7)	Renal cancer	Partial nephrectomy	Outcomes compared to prospectively maintained database Lower estimated blood loss was only statistically significant difference	Not reported
Wake et al. (15)	Case series (*n* = 10)	Renal cancer	Partial and Radical Nephrectomy	For all cases 3D printed models changed some aspect of the surgical approach initially planned from 2D imaging	$1000 10 h
Golab et al. (23)	Case series (*n* = 3)	Renal cancer	Partial nephrectomy	-Reported that rehearsal on simulation model accelerated the actual surgery: 1 patient not requiring renal ischemia and 2 with ischemic time <9 min -No control arm to confirm this	~100 Euros 7–8 h
Von Rundstedt et al. (36)	Feasibility study (*n* = 10)	Renal cancer	Partial nephrectomy	-Similar resection times between simulated rehearsal and procedure. -Similar enucleated tumor volume as well (36)	Not reported
Lee et al. (37)	Case series (*n* = 10)	Renal cancer	Partial nephrectomy	-Positive validation of models by urologists in understanding anatomy, preoperative surgical planning, intraoperative tumor localization -Also improved tumor localization by students (37)	Not reported
Komai et al. (14)	Case series (n = 10)	Renal cancer	Partial nephrectomy	-Reported models as consistent with intra-operative findings. -Reported resected tumor and margins nearly identical to model -No control group for comparison of surgical outcomes	$450-680 3-9 days
Zhang et al. (12)	Case series (n = 10)	Renal cancer	Partial nephrectomy	-Positive face validation of model by surgeons -High satisfaction of patients with the models	$150 3-4 days
Silberstein et al. (13)	Pilot study (n = 5)	Renal cancer	Partial nephrectomy	-Patients, families and trainees expressed improved comprehension -Surgeons referred to models during procedure -No comparative group for reported outcomes	Not reported
**SURGICAL PLANNING: PCNL, PROSTATE CANCER, KIDNEY TRANSPLANT, ADRENALECTOMY, FUNCTIONAL UROLOGY AND URETHRAL INJURY – 9**					
**PAPERS**					
Xu et al. (22)	Case series (*n* = 12)	Renal stones	PCNL	Simulated puncture at 3 different sites before choosing approach Comparative post-operative stone volume in models and patients	Not reported
Atalay et al. (8)	Case series (*n* = 5)	Renal stones	PCNL	Improved knowledge amongst residents of calyces, stone location and optimal entry calyx prior to procedure	$100 2 h
Golab et al. (23)	Case report (*n* = 1)	Renal stones	PCNL	Time to establish percutaneous access to kidney with surgical guide <3 min	Cost not reported 5 h 35 min
Kuroda et al. (38)	Case report (*n* = 1)	Renal stones	Ureteroscopy	Completed case with difficult anatomy achieving stone-free status without complication (38)	Not reported
Kusaka et al. (39)	Pilot Study (*n* = 2)	Renal transplant	Renal transplant	Reportedly helpful for recognizing anatomical features during procedure Pre-surgical simulation reportedly accurately mimicked the surgical procedure (39)	Not reported
Shin et al. (19)	Proof of concept (*n* = 5)	Prostate cancer	Robot assisted radical prostatectomy	3D models used as reference during surgery. Negative margins for all cases in spite of being high-risk cases	$500
Wang et al. (20)	Case series (*n* = 16)	Prostate cancer	Prostate biopsy	Higher positive biopsy rate for targeted biopsies using model Comparison to systematic biopsy only. Not compared to cognitive biopsy with imaging alone or template biopsy	Not reported
Srougi et al. (40)	Case report (*n* = 1)	Adrenal	Partial adrenalectomy	3D printed replica examined by surgeon before completing total left adrenalectomy and partial right adrenalectomy. Surgical outcomes described but no data or control group for comparison (40)	Not reported
Zhang et al. (12)	Case series (n =24)	Functional urology	Sacral neuromodulation	Reduced number of punctures Reduced puncture time Reduced X-ray exposure	$500

## Surgical Training

Gasior detailed 3D-printings use in improving understanding of complex cloacal anomalies for trainees and faculty surgeons alike (26). Tangible 3D-printed model demonstrated improved questionnaire-assessed understanding compared to 2D cloacagrams, rotatable 3D virtual models and 3D video animations.

Simulation with 3D-printed anatomical models has been a focus of research, offering patient-specific representation of pathology and tactile feedback. Simulation utilizing 3D-printing has been described for partial nephrectomy, pyeloplasty, PCNL, robot-assisted prostatectomy, robot-assisted kidney transplant, vasectomy reversal, and transurethral resection of the prostate (TURP).

Adams created “phantom” models of cadaveric kidneys using 3D-printed molds designed from CT imaging. These were compared to the original in ultrasound and CT appearances as well as hardness, elasticity, and tensile strength. Different materials were trialed and the water-based gel, Agarose proved most similar to human kidney tissue (27). Melnyk recently created poly-vinyl alcohol (PVA) kidney phantoms using 3D-printed casts derived from a patient with a 4.2 cm exophytic renal tumor (28). Renal vasculature and collecting system were constructed from PVA and models were tested against porcine equivalents in mechanical properties & flow characteristics of the simulated vasculature. The group also compared suture tension required to approximate renal parenchymal edges and the maximum tension at which suture tension tore through parenchyma. Testing identified 7% PVA models with a three freeze-thaw cycle as the formula best replicating porcine tissue. The models were set amongst fabricated peritoneum, abdominal fat, spleen, bowel and mesentry to form an immersive simulation of robot-assisted partial nephrectomy. Similarly, Choi created prostate phantoms for simulation of TURP using PVA, agar and hollow glass powder, testing compressive & elastic properties. Models with different agar percentages were compared to normal, cancerous, and hyperplastic prostate tissue (29) with the physical properties of these simulation models being demonstrated as adjustable.

Ghazi's PVA hydrogel models of the kidney and adjacent structures allowed immersion simulation of PCNL with experts (caseload >100) and novices (caseload <20) rating it highly in similarity to the real procedure and usefulness in training. The models' realism was further supported by significant superiority from experts compared to novices in mean fluoroscopy time, number of percutaneous access attempts and stone clearance (30). Cheung et al printed molds of renal anatomy with pelvic ureteric junction obstruction and cast silicone rubber models for low-cost, reusable simulation of laparoscopic pyeloplasty (31). The simulation was validated on a 5-point Likert scale, scoring 4.75 (± 0.29), 4.50 (± 0.41), and 4.38 (± 0.48) in overall impression, realism and handling, respectively. Shee used printed casts to mold a surrogate bladder neck and urethra from silicone for an *ex-vivo* simulation trainer for robotic vesicourethral anastomosis (32) with an average face validity rating of 8/10 and content validity of 10/10. Experts and trainees rated the simulation superior to digital virtual reality (VR) trainers and experts performed better than residents in the simulation. Uwechue developed a 3D-printed simulation model for vascular anastomosis during robot-assisted kidney transplant, highlighting potential training value even amongst experienced surgeons when learning new surgical techniques (33). Pinto reported an observed improvement in residents microsurgical suture time and quality on a vasectomy reversal model produced via 3D-printing (41). However, as Monda et al commented in their paper on partial nephrectomy simulation, future studies need to establish that improved performance on simulation models is associated with improvements in live surgery (42).

Simulation presents a reproducible and uniform means to assess trainee performance both subjectively and with quantitative metrics. The aforementioned TURP model by Choi used materials with different ultrasound echogenicity for the central and peripheral zones of the prostate so that the resected volume of each zone could be assessed (29). Qiu incorporated tactile sensors into 3D-printed prostate models able to calculate pressure forces applied to the model (43). Witthaus et al. constructed a simulation model for robot-assisted radical prostatectomy using a chemiluminescent dye-impregnated PVA hydrogel model of the prostate and a tension wire sensor incorporated within the neurovascular bundle (44). The tension wire provided quantitative measurement of tension applied to the NVB during nerve-sparing prostatectomy and the dye allowed assessment of the surgical margin. Further metrics included the urethrovesical anastomosis leak test and task-specific times. When compared against assessment via a Global Evaluative Assessment of Robotic Skills (GEARS) and Robotic Anastomosis Competency Evaluation (RACE), higher GEARS score tallied with lower exerted force on the NVB and higher RACE scores correlated with a lower UVA leak rate. Witthaus' paper highlighted novel ways in which 3D-printing and simulation can be designed to objectively assess trainees.

Parkhomenko et al aided surgical simulation in a different way by designing a 3D-printable, portable laparoscopic trainer with a reported production cost of $26.50 and assembly time of <45 min (4). Whilst conventional trainers were scored higher by trainees, all still reported it as useful and this study demonstrated how 3D-printable designs can easily be shared across institutions for immediate production. A summary of reviewed articles relating to surgical training is provided in Table 3.

**Table 3:** Articles relating to surgical training−17 papers.

**References**	**Paper type and case number**	**Area of urology**	**Application**	**Reported outcomes and limitations**	**Cost and time to produce model**
Gasior et al. (26)	Single case study (*n* = 1)	Pediatrics	Congenital anomalies	Increased understanding of anatomy as assessed by questionnaire Compared to inspection of 2D imaging, rotating 3D computer model and interactive 3D computer model.	Cost not reported 18 h
Melnyk et al. (28)	Simulation validation (n/a)	Renal cancer	Partial nephrectomy	7% polyvinyl alcohol at three freeze-thaw cycles found to best replicate mechanical properties of porcine tissue	$43.30 in material $60 in personnel $82 in consumables
Monda et al. (42)	Simulation validation (n/a)	Renal cancer	Partial nephrectomy	Silicone renal tumor model demonstrating face, content and construct validity Surgeons of higher training levels performed better on the model	$260 for molds $3.90 per model 2 h
Smektala et al. (23)	Technical note (n/a)	Renal cancer	Partial nephrectomy	Present steps for producing low-cost silicone renal models for partial nephrectomy simulation (55)	$14.4 for mold $7.4 per model
Knoedler et al. (56)	Model validation (n/a)	Renal cancer	Partial nephrectomy	Accuracy of the deduced nephrometry score was improved in trainees by 3D-printed models when compared to standard imaging (56)	Not reported
Porpiglia et al. (57)	Case series (*n* = 18)	Renal and prostate cancer	Partial nephrectomy and Radical prostatectomy	Positive face and content validity when assessed by surgical trainees (57)	Not reported
Shee et al. (32)	Simulation validation (n/a)	Prostate cancer	Robot assisted radical prostatectomy	Average face validity 8/10 Average content validity 10/10 Improved performance observed in experts in procedure compared to trainees	$80 for mold $5 silicone model $100 acrylic frame $10 labor per model
Qiu et al. (43)	Model validation (n/a)	Prostate	Not specified	Models with tissue-mimicking tactile sensation and behavior Sensors allowing quantitative measurement of pressure applied to model	Not reported
Witthaus et al. (44)	Simulation validation (n/a)	Prostate cancer	Robot assisted radical prostatectomy	Incorporated quantitative measures of performance into model of robot-assisted radical prostatectomy simulation model Demonstrated objective scoring systems (GEARS and RACE) as correlating well with quantitative outcome measures	-
Ghazi et al. (30)	Simulation validation (n/a)	Renal Stones	PCNL	Average face and content validity of 4.5/10 and 4.6/10 respectively Lower fluoroscopy time, number of puncture attempts for experts compared to trainees and also better stone clearance	-
Choi et al. (29)	Simulation validation (n/a)	Benign prostatic hyperplasia	TURP	Demonstrated adjustable compressive elastic properties of model Enabled quantitative evaluation of resection Electrocautery of model closely resembled the procedure on human tissue	Not reported
Cheung et al. (31)	Simulation validation (n/a)	PUJ obstruction	Pyeloplasty	Average scoring by urology fellows and faculty: Realism 4.50/5 Handling 4.38/5 Usability 3.6/5 (novices), 3.7/5 (experts) Aesthetics 3.5/5 (novices), 3.3/5 (experts)	~$100
Uwechue et al. (33)	Simulation validation (n/a)	Kidney transplant	Kidney transplant	Allowed bespoke immersion simulation of robot-assisted renal transplant	$1000
Pinto et al. (41)	Simulation validation (n/a)	Andrology	Vasectomy reversal	Measured performance in terms of completion time and objective performance checklist with an observed improvement on repeated use	Not reported
Parkhomenkho et al. (6)	Model validation	Laparoscopy	Laparoscopy	Designed a laparoscopic trainer which could be digitally shared and produced across institutions with reported low cost and assembly time. Scored lower than conventional trainers but still rated as useful	$26.50
Sweet (58)	Model validation (n/a)	All urology	All urology	Described the development process used by the Center of Research in Education and Simulation Technologies for several simulation models (58)	-
Adams et al. (27)	Model validation (n/a)	All renal	All renal	Demonstrated agar hydrogel models of the kidney as having physical properties most consistent with human tissue	Cost not reported 2 days

## Patient-Specific Prostheses & Bioprinting

Outside of urology 3D-printing has been used for customized orthopedic plate sizing and molding (45) as well as for titanium and ceramic patient-specific maxillofacial implants (46). 3D-printed implants in urology are limited by the unique mechanical and physiological functions of the genitourinary tract and further complicated by the need to be sterilisable. Patient-specific 3D-printed extravascular stent have been used to treat posterior nutcracker syndrome (47) (48) but these do not emulate any urological structure. CT imaging was used to design a custom stent, 3D-printed from a titanium alloy and laparoscopically sited around the retro-aortic left renal vein to prevent compression.

However, substitutional grafts within the genitourinary tract remain possible via bioprinting. Organ production from native tissue over a scaffold was demonstrated in the Vacanti mouse where a chondrocyte-seeded, ear-shaped scaffold was implanted beneath its skin (49). Atala similarly used urothelial and smooth muscle cells obtained from bladder biopsy to ‘grow' autologous tissue around a biodegradable bladder-shaped collagen scaffolds which were then successfully used for cystoplasty (50). Huang reported using 3D porous bacterial cellulose scaffolds seeded with rabbit lingual keratinocytes as a material for urethral reconstruction (51). These original cellulose scaffolds were not produced via 3D-printing but an integrated tissue-organ printer (ITOP) system was later used by Khang to print a porous, spiral scaffold dispersed with rabbit urothelial and smooth muscle cells within a fibrin hydrogel. This bioprinted urethra demonstrated mechanical properties equivalent to native rabbit urethra, with cells maintaining 80% viability at 7 days and demonstrating active proliferation (52). Versteegden also reported on collagen scaffolds produced via 3D-printing, reproducing the elasticity and shape-recovery of human urethral tissue (53). Yu investigated 3D-printed polycaprolactone (PCL) scaffolds and culture of human fibroblast cells for potential use as a surrogate for tunica albuginea (54) and Oh et al successfully cultured human aortic smooth muscle and umbilical vein endothelial cells over 3D-printed PCL scaffolds as a potential tissue-engineered corpus cavernosum graft (63).

Whilst these studies demonstrate how unique mechanical properties of the urinary tract can be emulated, a more significant development toward future autologous kidney replacement is the recent bioprinting of a renal proximal convoluted tubule. This involved printing a silicone gasket within a perfusable “3D tissue chip” and seeding it with immortalized human proximal tubular cells (61) forming a polarized epithelium, functional as a barrier and damaged by known nephrotoxins. A summary of reviewed articles relating to patient-specific prostheses and bioprinting is provided in Table 4.

**Table 4:** Articles relating to patient-specific prostheses and bioprinting−8 papers.

**References**	**Paper type and case number**	**Area of urology**	**Application**	**Reported outcomes and limitations**
Kim et al. (59)	Lab	Bladder cancer	Histological and pharmaceutical	Higher cancer cell proliferation in 3D models with higher cell-cell interactions (59) Showed that medication effects were more exaggerated in 2D culture compared to 3D
Oh et al. (60)	Lab	Andrology	Cavernosal graft	Cells cultured over a 3D printed scaffold remained viable and proliferated
Yu et al. (61)	Lab	Andrology	Tunica graft	Cells remained viable and proliferated forming cell sheets around the scaffold with cellular bridges
Versteegden et al. (53)	Lab	Andrology	Urethral graft	Cells cultured over 3D-printed collagen star-shaped scaffold Scaffold mimicked the dynamics of the human urethra
Zhang et al. (62)	Lab	Andrology	Urethral graft	Bioprinted urethra with 80% cell viability at 7 days and mechanical properties equivalent to native rabbit urethra
Huang et al. (51)	Lab	Andrology	Urethral graft	Reconstructed rabbit urethra with 3D cellulose scaffold seeded with lingual keratinocytes At 3 months, seeded scaffold maintained urethral caliber and exhibited epithelial regeneration
Wang et al. (20)	Case series (n =17)	Renal	Nutcracker syndrome treatment	CT imaging used to 3D print, individualized extravascular stents to treat posterior nutcracker syndrome Implanted without complication Stable sent position at follow up
Guo et al. (48)	Case report	Renal	Nutcracker syndrome treatment	Single case of posterior nutrcracker syndrome treated with 3D printed, patient-specific extravascular stent

## Surgical Tools

Park published *in vitro* test results for a 3D-printed anti-reflux ureteric stent (60) whilst Junco et al created ureteric stents and laparoscopic trocars via 3D-printing, tested in porcine and cadaveric models. Junco was successful in producing 9F and 12F diameter stents which were deployable via a 0.035 guidewire but the smaller 7F stent did not allow passage of a guidewire and they were unable to print a stent with a tapered end. The 3D-printed trocars maintained pneumoperitoneum and allowed instrument passage but formed larger superficial skin defects than Karl Storz and Ethicon equivalents (64). Issues of biocompatibility, sterility, durability, tensile strength, and stent encrustation were not evaluated or addressed in the study.

Canvasser's pilot study of 3D-printed surgical clips proved inefficacious as they broke, failed to close and leaked more than commercially available alternatives (65). Rankin's military skin retractors 3D-printed using polylactic acid (PLA) filament were sterilisable via glutaraldehyde protocols and freshly printed retractors were sterile on bacterial testing via polymerase chain reaction (PCR). When stressed until fracture they could tolerate 13.6 kg of tangential force with no significant change after glutaraldehyde sterilization. Whilst insufficient for retraction of the abdominal wall, skin flaps or for orthopedic procedures this was fit for purpose as a skin retractor and low cost ($2.77 per retractor compared to the $23.48 stainless steel equivalent) (66). The cost of disposable equipment due to packaging, sterilization, transportation & storage could be overcome with on-site printing of tools and advantages would be greater in developing countries, where access to surgical equipment is limited by cost and transport. A summary of reviewed articles relating to surgical tools is provided in Table 5.

**Table 5:** Articles relating to surgical tools−5 papers.

**References**	**Paper type**	**Area of urology**	**Application**	**Reported outcomes and limitations**
Del Junco et al. (64)	Lab	Endourology	Ureteric stent	3D printed stents in *ex-vivo* models showed comparable flow rate characteristics to contemporary stents Unable to produce tapered ends
Park et al. (60)	Lab	Endourology	Ureteric stent	*In vitro* study of 3D-printed antireflex stent showing effect prevention of backflow
Del Junco et al. (64)	Lab	Endourology/Laparoscopy	Ureteric stents and laparoscopy trocars	Able to 3D-print ureteric stents introduced by seldinger technique but not for smaller stent sizes (7F) Functional trocars but produced larger superficial skin defects than contemporary products
Canvasser et al. (65)	Lab	Laparoscopy	Surgical clips	Clips had a fracture rate of 54% Only 23 of 50 clips closed These leaked at a mean pressure of 20.7 kPa. No commercial clips broke or leaked
Rankin et al. (66)	Lab	Open surgery	Surgical retractors	3D-printed sterile Polylactic Acid filament retractors which where sterilisable Low tangential strength limiting use to skin retraction

## Novel Uses

Morimoto described a virtual reality design interface and 3D-printed concentric tube robot produced by this method. The surgeon-designed robot extended to curve beneath the 12th rib, into the kidney and upwards to the tumor (67). This prototyped a potential surgical instrument for tumor ablation but the study's focus was the interface which incorporated patient imaging and allowed the design and 3D-printing of patient-specific equipment by an individual surgeon.

3D-printed molds were used in two studies to standardize histological sampling of malignant renal and prostate specimens against imaging to validate the accuracy of imaging in identifying tumor location and likely histological findings (62, 68, 69). Antonelli used renal models to simulate PCNL and test a new “PercSac” device for capturing stones and fragments during the procedure (70).

Tse et al developed the “Endockscope” incorporating a cordless, light-emitting diode (LED) light source and a 3D-printed attachment for connecting smartphones to the flexible cystoscope eyepiece (71). Testing different smartphones against a Storz HD camera and xenon light, all were inferior to the standard system in image quality, brightness and color but combination with the Samsung S8 was uniformly rated by faculty urologists as acceptable for use.

## Future Directions

Proposed applications for 3D printing within urology are expanding, with particular focus on tangible models of patient anatomy to enhance patient understanding, surgical planning and simulation training. The rapid increase in published research has been accompanied by related review articles. Smith and Dasgupta (72) reviewed applications within urological training whilst Checcuchi (73) provides a non-systematic review of both virtual and printed 3D models' utilization in robotic urological surgery. This review gives a concise overview of all reported uses of 3D printing across urology. Both Cacciamani's and Chen's systematic reviews (74, 75) provide a comprehensive exploration of published applications with expanded analyses of setup and manufacturing costs. However, we provide a more contemporary literature search and as a mini-review, a rapidly accessible outline of both the promising existing evidence and shortcomings in linking this to an improved service; Does elevating patient understanding reduce anxiety and litigation or improve recovery? Can inspection of or simulation with a patient-specific model improve surgical outcomes? Does simulation training with tangible facsimiles improve surgical training and with superiority to existing simulation models? These answers remain unproven and should underpin future research.

Regardless of how many prove durably useful, the scope of novel developments within urology showcase the freedom to innovate and rapidly prototype afforded by 3D printing. Whilst bioprinting promises major potential advances in patient-specific grafts in the future, we believe rapid prototyping coupled with network sharing and development of ideas is why 3D-printing will remain pivotal in ongoing surgical innovation. Table 6 provides an overview of all reported clinical applications and evidence limitations in the reviewed articles.

**Table 6:** Summary of clinical applications and evidence limitations.

**Proposed clinical application of 3D printing**	**Limitations of evidence**
**PATIENT EDUCATION**	
Explanation with patient-specific 3D-printed models can - Improve questionnaire-assessed patient understanding prior to partial nephrectomy, PCNL, and radical prostatectomy	No comparison to explanation with a general renal tumor/kidney stone/prostate model. Not conclusive that patient-specific models are required to improve understanding.
- Improve patient understanding compared to routine 2D imaging	Reported benefits of explanation with a 3D model confounded by duplication of the explanation process
- Improve patient understanding compared to other novel models; augmented reality and 3D computer models	Entailed a second explanation so benefit over standard explanation alone is confounded
- Improve patient rated comfort with the planned surgical intervention	More evidence required linking improved patient understanding with other measurable outcomes: e.g., patient anxiety, post-op recovery, and length of stay
**SURGICAL PLANNING**	
Inspecting tangible patient-specific models can be used to inform and optimize surgical approach	Low volume evidence that the surgical outcomes are improved significantly with many studies lacking a control arm
Simulated rehearsal of procedures with patient-specific models may reduce operating time and improve surgical outcomes	Evidence of improved outcomes is so far limited to estimated blood loss and warm ischemic time for partial nephrectomy. Small case series
Reported higher positive targeted biopsy rate if MR identified prostate lesions are presented in 3D models	Requires cost comparison of patient-specific models vs. fusion targeted biopsy
Surgical guides may improve time to PCNL access	The quicker nephroscopic access is unlikely to be as useful for uncomplicated cases due to the extra time required designing the guide. Automated design of guides for optimal access would be invaluable.
Using surgical guides for sacral-neuromodulation tine insertion can reduce punctures, procedure time, fluoroscopy time and optimize tine positioning.	Small cohort studies. Similarly automation of guide design would be invaluable
**SURGICAL TRAINING**	
Understanding of complex anatomical pathology can be improved with 3D printed models	Single case study
The physical properties of simulation models are adjustable to approximate the behavior of different tissues	Reviewed studies compared physical properties of models to porcine equivalents
Phantoms of patient-specific anatomy/pathology made via 3D printed casts can be used to simulate surgery (e.g., TURP, partial nephrectomy, prostatectomy, PCNL and vasectomy)	Further research is required to link practice on simulation models to improved surgical performance
Simulation with replicable anatomy allows a more standardized means of trainee assessment further aided by quantifiable metrics in simulation models	Requires further assessment of expert performance and tissue behaviors to interpret quantifiable metrics
**SURGICAL TOOLS**	
Reduced cost and waste from disposable surgical tools by producing as-required tools	Tools produced to date are inferior to existing alternatives in function and suitability for sterilization
**PATIENT-SPECIFIC PROSTHESES AND BIOPRINTING**	
Future prospect of autologous grafts for urethra, corpora and even the kidney with successful generation of tissues exhibiting similar mechanical and physiological properties	Very early stages of research.

## Author Contributions

DM read the referenced articles and wrote the first draft of the manuscript. AB and ML reviewed and edited the manuscript and approved the final version of the manuscript.

## Conflict of Interest

The authors declare that the research was conducted in the absence of any commercial or financial relationships that could be construed as a potential conflict of interest.
